# The approximation of Laplace-Stieltjes transforms with finite order

**DOI:** 10.1186/s13660-017-1441-9

**Published:** 2017-07-12

**Authors:** Hong Yan Xu, San Yan Liu

**Affiliations:** 1 School of Mathematics and Statistics, Xidian University, Xi’an, Shaanxi 710126 China; 2 Department of Informatics and Engineering, Jingdezhen Ceramic Institute, Jingdezhen, Jiangxi 333403 China

**Keywords:** 44A10, 30D15, irregular growth, Laplace-Stieltjes transform, approximate, error

## Abstract

In this paper, we study the irregular growth of an entire function defined by the Laplace-Stieltjes transform of finite order convergent in the whole complex plane and obtain some results about *λ*-lower type. In addition, we also investigate the problem on the error in approximating entire functions defined by the Laplace-Stieltjes transforms. Some results about the irregular growth, the error, and the coefficients of Laplace-Stieltjes transforms are obtained; they are generalization and improvement of the previous conclusions given by Luo and Kong, Singhal and Srivastava.

## Introduction

Dirichlet series

1 $$ f(s)=\sum_{n=1}^{\infty }a_{n}e^{\lambda_{n}s}, \quad s=\sigma +it, $$

where

2 $$ 0\leq \lambda_{1}< \lambda_{2}< \cdots < \lambda_{n}< \cdots , \qquad \lambda _{n}\rightarrow \infty\quad \mbox{as } n\rightarrow \infty ; $$

$s=\sigma +it$ (*σ*, *t* are real variables), $a_{n}$ are nonzero complex numbers. When $a_{n}$, $\lambda_{n}$, *n* satisfy some conditions, the series (1) is convergent in the whole plane or the half-plane, that is, $f(s)$ is an analytic function or entire function in the whole plane or the half-plane. In the past few decades, many mathematicians studied the growth and value distribution of the analytic (entire) function defined by Dirichlet series and obtained lots of interesting results (see [1–9]).

As we know, Dirichlet series is regarded as a special example of the Laplace-Stieltjes transform. The Laplace-Stieltjes transform, named for Pierre-Simon Laplace and Thomas Joannes Stieltjes, is an integral transform similar to the Laplace transform. For real-valued functions, it is the Laplace transform of a Stieltjes measure, however it is often defined for functions with values in a Banach space. It can be used in many fields of mathematics, such as functional analysis, and certain areas of theoretical and applied probability.

For the Laplace-Stieltjes transforms,

3 $$ G(s)= \int_{0}^{+\infty }e^{-sx}\,d\alpha (x), \quad s= \sigma +it, $$

where $\alpha (x)$ is a bounded variation on any finite interval $[0,Y]$ ($0< Y<+\infty$), and *σ* and *t* are real variables. Let

$$ B_{n}^{*}=\sup_{\lambda_{n}< x\leq \lambda_{n+1},-\infty < t< +\infty } \biggl\vert \int_{\lambda_{n}}^{x}e^{-ity}\,d\alpha (y)\biggr\vert , $$

where the sequence $\{\lambda_{n}\}_{n=1}^{\infty }$ satisfies (2) and

4 $$ \limsup_{n\rightarrow +\infty }(\lambda_{n+1}- \lambda_{n})=h< +\infty. $$

In 1963, Yu [10] proved the Valiron-Knopp-Bohr formula of the associated abscissas of bounded convergence, absolute convergence, and uniform convergence of Laplace-Stieltjes.

### Theorem A

*Suppose that Laplace*-*Stieltjes transforms* (3) *satisfy* (2), (4) *and* $\limsup_{n\rightarrow +\infty }\frac{ \log n}{\lambda_{n}}<+\infty $, *then*

$$ \limsup_{n\rightarrow +\infty }\frac{\log B_{n}^{\ast }}{\lambda_{n}} \leq \sigma_{u}^{G} \leq \limsup_{n\rightarrow +\infty }\frac{\log B _{n}^{\ast }}{\lambda_{n}}+\limsup_{n\rightarrow +\infty } \frac{ \log n}{\lambda_{n}}, $$

*where* $\sigma_{u}^{F}$ *is called the abscissa of uniform convergence of* $F(s)$.

Moreover, Yu [10] first introduced the maximal molecule $M_{u}(\sigma ,G)$, the maximal term $\mu (\sigma ,G)$ and the Borel line, and the order of analytic functions represented by Laplace-Stieltjes transforms convergent in the complex plane. After his works, considerable attention has been paid to the growth and value distribution of the functions represented by the Laplace-Stieltjes transform convergent in the half-plane or the whole complex plane in the field of complex analysis (see [11–15]).

In 2012, Luo and Kong [16] studied the following form of Laplace-Stieltjes transform:

5 $$ F(s)= \int_{0}^{+\infty }e^{sx}\,d\alpha (x), \quad s= \sigma +it, $$

where $\alpha (x)$ is stated as in (3), and $\{\lambda_{n}\}$ satisfies (2),(4). Set

$$ A_{n}^{*}=\sup_{\lambda_{n}< x\leq \lambda_{n+1},-\infty < t< +\infty } \biggl\vert \int_{\lambda_{n}}^{x}e^{ity}\,d\alpha (y)\biggr\vert . $$

By using the same argument as in [10], we can get a similar result about the abscissa of uniform convergence of $F(s)$ easily. If

6 $$ \limsup_{n\rightarrow +\infty }\frac{\log n}{\lambda_{n}}=D< \infty , \qquad \limsup_{n\rightarrow +\infty }\frac{\log A_{n}^{\ast }}{\lambda_{n}}=- \infty , $$

by (2), (4) and Theorem 1, one can get that $\sigma_{u}^{F}=+\infty $, $i.e$., $F(s)$ is an entire function.

Set

$$ M(\sigma ,F)=\sup_{-\infty < t< +\infty }\bigl\vert F(\sigma +it)\bigr\vert ,\qquad M _{u}(\sigma ,F)=\sup_{0< x< +\infty ,-\infty < t< +\infty }\biggl\vert \int_{0} ^{x}e^{(\sigma +it)y}\,d\alpha (y)\biggr\vert $$

and

$$ \mu (\sigma ,F)=\max_{n\in N}\bigl\{ A_{n}^{*}e^{\lambda_{n}\sigma } \bigr\} ( \sigma < +\infty ),\quad N(\sigma ,F)=\max \bigl\{ \lambda_{n}: ~A_{n}^{*}e ^{\lambda_{n}\sigma }=\mu (\sigma ,F)\bigr\} . $$

Since $M(\sigma ,F)$ and $M_{u}(\sigma ,F)$ tend to +∞ as $\sigma \rightarrow +\infty $, in order to estimate the growth of $F(s)$ more precisely, we will adapt some concepts of order, lower order, type, lower type as follows.

### Definition 1.1

If Laplace-Stieltjes transform (5) satisfies $\sigma_{u}^{F}=+\infty $ (the sequence $\{\lambda_{n}\}$ satisfies (2), (4), and (6)) and

$$ \limsup_{\sigma \rightarrow +\infty }\frac{\log^{+}\log^{+}M_{u}( \sigma ,F)}{\sigma }=\rho , $$

we call $F(s)$ of order *ρ* in the whole plane, where $\log^{+}x= \max \{\log x,0\}$. If $\rho \in (0,+\infty )$, we say that $F(s)$ is an entire function of finite order in the whole plane. Moreover, the lower order of $F(s)$ is defined by

$$ \liminf_{\sigma \rightarrow +\infty }\frac{\log^{+}\log^{+}M_{u}( \sigma ,F)}{\sigma }=\lambda . $$

### Remark 1.1

We say that $F(s)$ is of the regular growth, when $\rho =\lambda $, and $F(s)$ is of the irregular growth, when $\rho \neq \lambda $.

### Definition 1.2

If Laplace-Stieltjes transform (5) satisfies $\sigma_{u}^{F}=+\infty $ (the sequence $\{\lambda_{n}\}$ satisfies (2), (4), and (6)) and is of order *ρ* ($0<\rho <\infty$), then we define the type and lower type of L-S transform $F(s)$ as follows:

$$ \limsup_{\sigma \rightarrow +\infty }\frac{\log^{+}M_{u}(\sigma ,F)}{e ^{\sigma \rho }}=T,\qquad \liminf _{\sigma \rightarrow +\infty }\frac{ \log^{+}M_{u}(\sigma ,F)}{e^{\sigma \rho }}=\tau . $$

### Remark 1.2

The purpose of the definition of type is to compare the growth of class functions which all have the same order. For example, let $f(s)=e^{e ^{s}}$, $g(s)=e^{e^{2s}}$, by a simple computation, we have $\rho (f)=1= \rho (g)$, but $T(f)=1$ and $T(g)=\infty $. Thus, we can see that the growth of $g(s)$ is faster than $f(s)$ as $\vert s \vert \rightarrow +\infty $.

## Results and discussion

Recently, many people studied some problems on analytic functions defined by the Laplace-Stieltjes transforms and obtained a number of interesting results. Kong, Sun, Huo and Xu investigated the growth of analytic functions with kinds of order defined by the Laplace-Stieltjes transforms (see [16–22]), and Shang, Gao, and Sun investigated the value distribution of such functions (see [23–26]). From these references, we get the following results.

### Theorem 2.1

*If Laplace*-*Stieltjes transform* (5) *satisfies* $\sigma_{u}^{F}=+\infty $ (*the sequence* $\{\lambda_{n}\}$ *satisfies* (2), (4), *and* (6)), *and is of order* *ρ* ($0<\rho <\infty$) *and of type* *T*, *then*

$$ \rho =\limsup_{n\rightarrow +\infty }\frac{\lambda_{n}\log \lambda _{n}}{-\log A_{n}^{*}}, \qquad T=\limsup _{n\rightarrow +\infty }\frac{ \lambda_{n}}{\rho e}\bigl(A_{n}^{*} \bigr)^{\frac{\rho }{\lambda_{n}}}. $$

*Furthermore*, *if* $F(s)$ *is of the lower order* *λ* *and the lower type* *τ*, *and* $\lambda_{n}\sim \lambda_{n+1}$ *and the function*

$$ \psi (n)=\frac{\log A^{*}_{n}-\log A^{*}_{n+1}}{\lambda_{n+1}-\lambda _{n}} $$

*forms a non*-*decreasing function of* *n* *for* $n>n_{0}$, *then we have*

$$ \lambda =\liminf_{n\rightarrow +\infty }\frac{\lambda_{n}\log \lambda _{n}}{-\log A_{n}^{*}}, \qquad \tau = \liminf _{n\rightarrow +\infty }\frac{ \lambda_{n}}{\rho e}\bigl(A_{n}^{*} \bigr)^{\frac{\rho }{\lambda_{n}}}. $$

From Definition 1.2, a natural question to ask is: What happened if $e^{\sigma \rho }$ is replaced by $e^{\lambda \sigma }$ in the definition of lower type when $\rho \neq \lambda $? We are going to consider this question.

### Definition 2.1

If Laplace-Stieltjes transform (5) satisfies $\sigma_{u}^{F}=+\infty $ (the sequence $\{\lambda_{n}\}$ satisfies (2), (4), and (6)), and is of order *ρ* ($0<\rho <\infty$) and of the lower order *λ* ($0<\lambda <\infty$), if $\lambda \neq \rho $, and

$$ \liminf_{\sigma \rightarrow +\infty }\frac{\log^{+}M_{u}(\sigma ,F)}{e ^{\sigma \lambda }}=\tau_{\lambda }, $$

we say that $\tau_{\lambda }$ is the *λ*-type of $F(s)$.

### Remark 2.1

Obviously, $\tau_{\lambda }\geq \tau $ and $\tau_{\lambda }=\tau $ as $\rho =\lambda $. But we cannot confirm whether $\tau_{\lambda } \geq T$ or $\tau_{\lambda }\leq T$.

The following results are the main theorems of this paper.

### Theorem 2.2

*If Laplace*-*Stieltjes transform* (5) *satisfies* $\sigma_{u}^{F}=+\infty $ (*the sequence* $\{\lambda_{n}\}$ *satisfies* (2), (4), *and* (6)), *and is of order* *ρ* *and of the lower order* *λ*, $0\leq \lambda \neq \rho <\infty $, *then we have*

7 $$ \liminf_{\sigma \rightarrow \infty }\frac{\log M(\sigma ,F)}{e^{ \rho \sigma }}= \liminf _{\sigma \rightarrow \infty }\frac{\log \mu ( \sigma ,F)}{e^{\rho \sigma }}=0, $$

*and*

8 $$ \liminf_{\sigma \rightarrow \infty }\frac{N(\sigma ,F)}{e^{\rho \sigma }}=0. $$

### Theorem 2.3

*If Laplace*-*Stieltjes transform* (5) *satisfies* $\sigma_{u}^{F}=+\infty $ (*the sequence* $\{\lambda_{n}\}$ *satisfies* (2), (4), *and* (6)), *and is of order* *ρ* *and of the lower order* *λ*, $0< \lambda \neq \rho <\infty $, *type* *T*, *λ*-*type* $\tau_{\lambda }$,

$$ \limsup_{\sigma \rightarrow +\infty }\frac{N(\sigma ,F)}{e^{\rho \sigma }}=H, \qquad \liminf _{\sigma \rightarrow +\infty }\frac{N(\sigma ,F)}{e^{\rho \sigma }}=h, $$

*and let*

$$ T_{\rho }(\sigma ,F)=\frac{\log \mu (\sigma ,F)}{\exp (\rho \sigma )},\qquad T_{\lambda }(\sigma ,F)= \frac{\log \mu (\sigma ,F)}{\exp (\lambda \sigma )}, $$

*then we have*

9 $$\begin{aligned}& H-\rho T\leq \limsup_{\sigma \rightarrow +\infty }T'_{\rho }( \sigma ,F) \leq H, \end{aligned}$$

10 $$\begin{aligned}& -\infty \leq \liminf_{\sigma \rightarrow +\infty }T'_{\lambda }( \sigma ,F)\leq h-\lambda \tau_{\lambda } \end{aligned}$$

*for almost all values of* $\sigma >\sigma_{0}$, *where* $T'_{\rho }( \sigma )$ *and* $T'_{\lambda }(\sigma )$ *are the derivatives of* $T_{\rho }(\sigma )$ *and* $T_{\lambda }(\sigma )$ *with respect to* *σ*.

### Theorem 2.4

*If Laplace*-*Stieltjes transform* (5) *satisfies* $\sigma_{u}^{F}=+\infty $ (*the sequence* $\{\lambda_{n}\}$ *satisfies* (2), (4), *and* (6)), *and is of the lower order* *λ* ($0\leq \lambda \neq \rho <\infty$), *if* $\lambda_{n}\sim \lambda_{n+1}$, *then*

11 $$ \tau_{\lambda }\geq \liminf_{n\rightarrow \infty } \biggl( \frac{\lambda _{n}}{e\lambda } \biggr) \bigl(A^{*}_{n} \bigr)^{\frac{\lambda }{\lambda_{n}}} \quad (0\leq \tau_{\lambda }\leq \infty ). $$

*Furthermore*, *there exists a positive integer* $n_{0}$ *such that*

$$ \psi (n)=\frac{\log A^{*}_{n}-\log A^{*}_{n+1}}{\lambda_{n+1}-\lambda _{n}} $$

*forms a non*-*decreasing function of* *n* *for* $n>n_{0}$, *then we have*

12 $$ \tau_{\lambda }= \liminf_{n\rightarrow \infty } \biggl( \frac{\lambda _{n}}{e\lambda } \biggr) \bigl(A^{*}_{n} \bigr)^{\frac{\lambda }{\lambda_{n}}} \quad (0\leq \tau_{\lambda }\leq \infty ). $$

We denote by $\overline{L}_{\beta }$ the class of all the functions $F(s)$ of the form (5) which are analytic in the half-plane $\Re s<\beta$ ($-\infty <\beta <\infty$) and the sequence $\{\lambda _{n}\}$ satisfies (2) and (4); and we denote by $L_{\infty }$ the class of all the functions $F(s)$ of the form (5) which are analytic in the half-plane $\Re s<+\infty $ and the sequence $\{\lambda_{n}\}$ satisfies (2), (4), and (6). Thus, if $-\infty <\beta <+\infty $ and $F(s) \in \overline{L}_{\beta }$, then $F(s)\in L_{\infty }$. If Laplace-Stieltjes transform (5) $A^{*}_{n}=0$ for $n\geq k+1$ and $A^{*}_{n}\neq 0$, then $F(s)$ will be called an exponential polynomial of degree *k* usually denoted by $p_{k}$, $i.e$., $p_{k}(s)= \int^{\lambda_{k}}_{0}\exp (sy)\,d\alpha (y)$. When we choose a suitable function $\alpha (y)$, the function $p_{k}(s)$ may be reduced to a polynomial in terms of $\exp (s\lambda_{i})$, that is, $\sum_{i=1} ^{k}b_{i}\exp (s\lambda_{i})$.

For $F(s)\in \overline{L}_{\beta }$, $-\infty <\beta <+\infty $, we denote by $E_{n}(F,\beta )$ the error in approximating the function $F(s)$ by exponential polynomials of degree *n* in uniform norm as

$$ E_{n}(F,\beta )=\inf_{p\in \Pi_{n}}\Vert F-p \Vert _{\beta }, \quad n=1,2,\ldots , $$

where

$$ \Vert F-p \Vert _{\beta }=\max_{-\infty < t< +\infty }\bigl\vert F( \beta +it)-p(\beta +it) \bigr\vert . $$

In this paper, we will further investigate the relation between $E_{n}(F,\beta )$ and the growth of an entire function defined by the L-S transform with irregular growth. It seems that this problem has never been treated before. Our main result is as follows.

### Theorem 2.5

*If the Laplace*-*Stieltjes transform* $F(s)\in L_{\infty }$ *and is of lower order* *λ* ($0\leq \lambda \neq \rho <\infty$), *if* $\lambda_{n}\sim \lambda_{n+1}$, *then for any real number* $-\infty < \beta <+\infty $, *we have*

13 $$ \tau_{\lambda }\geq \liminf_{n\rightarrow \infty } \biggl( \frac{\lambda _{n}}{e\lambda } \biggr) \bigl(E_{n-1}(F,\beta )\exp (-\beta \lambda_{n})\bigr)^{\frac{ \lambda }{\lambda_{n}}}\quad (0\leq \tau_{\lambda }\leq \infty ). $$

*Furthermore*, *there exists a positive integer* $n_{0}$ *such that*

$$ \psi_{1}(n)=\frac{\log A^{*}_{n}-\log A^{*}_{n+1}}{\lambda_{n+1}- \lambda_{n}} $$

*forms a non*-*decreasing function of* *n* *for* $n>n_{0}$, *then we have*

$$ \tau_{\lambda }= \liminf_{n\rightarrow \infty } \biggl( \frac{\lambda _{n}}{e\lambda } \biggr) \bigl(E_{n-1}(F,\beta )\exp (-\beta \lambda_{n}) \bigr)^{\frac{ \lambda }{\lambda_{n}}}\quad (0\leq \tau_{\lambda }\leq \infty ), $$

*i*.*e*.,

14 $$ \exp (\beta \lambda )e\lambda \tau_{\lambda }= \liminf _{n\rightarrow \infty }\lambda_{n}\bigl(E_{n-1}(F,\beta ) \bigr)^{\frac{ \lambda }{\lambda_{n}}}. $$

## Conclusions

From Theorems 2.2-2.5, we can see that the growth of Laplace-Stieltjes transforms is investigated under the assumption $\rho \neq \lambda $, and that some theorems about the *λ*-lower type $\tau_{\lambda }$, $\lambda_{n}$, $A_{n}^{*}$, and *λ* are obtained. In addition, we also study the problem on the error in approximating entire functions defined by the Laplace-Stieltjes transforms. This project is a new issue of Laplace-Stieltjes transforms in the field of complex analysis. Our results are generalization and improvement of the previous conclusions given by Luo and Kong [16, 27], Singhal and Srivastava [28].

## Methods

### Proofs of Theorems 2.2 and 2.3

To prove the above theorems, we require the following lemmas.

#### Lemma 4.1

see [27], Lemma 2.1

*If the L*-*S transform* $F(s)\in L_{ \infty }$, *for any* $\sigma (-\infty <\sigma <+\infty )$ *and* $\varepsilon (>0)$, *we have*

$$ \frac{1}{2}\mu (\sigma ,F)\leq M_{u}(\sigma ,F)\leq C\mu \bigl((1+2\varepsilon )\sigma ,F\bigr), $$

*where* *C* *is a constant*.

#### Lemma 4.2

see [16], Lemma 2.2

*If the L*-*S transform* $F(s)\in L_{ \infty }$, *then we have*

$$ \log \mu (\sigma ,F)=\log \mu (\sigma_{0},F)+ \int_{\sigma_{0}}^{ \sigma }N(t,F)\,dt $$

*for* $\sigma_{0}>0$.

#### The proof of Theorem 2.2

Since $\rho >\lambda >0$ and $F(s)$ is of the lower order *λ*, that is,

15 $$ \lambda =\liminf_{\sigma \rightarrow +\infty }\frac{\log \log M_{u}( \sigma ,F)}{\sigma }, $$

for any small $\varepsilon (0<\varepsilon <\rho -\lambda )$, it follows from (15) that there exists a constant $\sigma_{0}$ such that, for $\sigma >\sigma_{0}$,

16 $$ \log M_{u}(\sigma ,F)>\exp \bigl\{ (\lambda -\varepsilon )\sigma \bigr\} , $$

and there exists a sequence $\{\sigma_{k}\}$ tending to +∞ such that

17 $$ \log M_{u}(\sigma_{k},F)< \exp \bigl\{ ( \lambda +\varepsilon )\sigma_{k}\bigr\} . $$

Since $0<\varepsilon <\rho -\lambda $, it follows from (16) and (17) that

18 $$ \liminf_{\sigma \rightarrow +\infty }\frac{\log M_{u}(\sigma ,F)}{ \exp (\rho \sigma )}=0. $$

From Lemmas 4.1 and 4.2, we have

$$ \rho =\limsup_{\sigma \rightarrow +\infty }\frac{\log \log M_{u}( \sigma ,F)}{\sigma } =\limsup _{\sigma \rightarrow +\infty }\frac{ \log \log \mu (\sigma ,F)}{\sigma } =\limsup_{\sigma \rightarrow + \infty } \frac{\log N(\sigma ,F)}{\sigma } $$

and

$$ \lambda =\liminf_{\sigma \rightarrow +\infty }\frac{\log \log M_{u}( \sigma ,F)}{\sigma } =\liminf _{\sigma \rightarrow +\infty }\frac{ \log \log \mu (\sigma ,F)}{\sigma } =\liminf_{\sigma \rightarrow + \infty } \frac{\log N(\sigma ,F)}{\sigma }. $$

Thus, similar to the process of (18), we can easily prove

$$ \liminf_{\sigma \rightarrow +\infty }\frac{\log \mu (\sigma ,F)}{ \exp (\rho \sigma )}= \liminf_{\sigma \rightarrow +\infty } \frac{N( \sigma ,F)}{\exp (\rho \sigma )}=0. $$

Hence, this completes the proof of Theorem 2.2.

#### The proof of Theorem 2.3

From Lemma 4.2, it follows that

19 $$ \limsup_{\sigma \rightarrow +\infty }\frac{\int_{\sigma_{0}}^{\sigma }N(t,F)\,dt}{e^{\rho \sigma }} =\limsup _{\sigma \rightarrow +\infty }\frac{ \log \mu (\sigma ,F)}{e^{\rho \sigma }}= \limsup_{\sigma \rightarrow +\infty }T_{\rho }( \sigma ,F)=T $$

and

20 $$ \liminf_{\sigma \rightarrow +\infty }\frac{\int_{\sigma_{0}}^{\sigma }N(t,F)\,dt}{e^{\lambda \sigma }} =\liminf _{\sigma \rightarrow +\infty }\frac{\log \mu (\sigma ,F)}{e^{\lambda \sigma }}= \liminf_{\sigma \rightarrow +\infty }T_{\lambda }( \sigma ,F)= \tau_{\lambda }. $$

Dividing two sides of the equality in Lemma 4.2 by $e^{\rho \sigma }$ and differentiating it with respect to *σ*, for almost all values $\sigma >\sigma_{0}$, we have

21 $$ T'_{\rho }(\sigma ,F)=-\rho \frac{\log \mu (\sigma_{0},F)}{e^{\rho \sigma }}-\frac{\rho }{e^{\rho \sigma }} \int_{\sigma_{0}}^{\sigma }N(t,F)\,dt+\frac{N( \sigma ,F)}{e^{\rho \sigma }}. $$

On the basis of the assumptions of Theorem 2.3, taking lim sup in (21) when $\sigma \rightarrow +\infty $, from Theorem 2.2 and (19), we get (9) easily.

Similarly, dividing two sides of the equality in Lemma 4.2 by $e^{\lambda \sigma }$ and differentiating it with respect to *σ*, for almost all values $\sigma >\sigma_{0}$,

22 $$ T'_{\lambda }(\sigma ,F)=-\lambda \frac{\log \mu (\sigma_{0},F)}{e ^{\lambda \sigma }}-\frac{\lambda }{e^{\lambda \sigma }} \int_{\sigma _{0}}^{\sigma }N(t,F)\,dt+\frac{N(\sigma ,F)}{e^{\lambda \sigma }}. $$

On the basis of the assumptions of Theorem 2.3, taking lim inf in (22) when $\sigma \rightarrow +\infty $, from Theorem 2.1 and (20), we get (10) easily.

Thus, this completes the proof of Theorem 2.3.

### The proof of Theorem 2.4

Let

$$ \vartheta =\liminf_{n\rightarrow +\infty }\frac{\lambda_{n}}{e\lambda } \bigl(A_{n}^{*}\bigr)^{\frac{\lambda }{\lambda_{n}}}\quad (0< \vartheta < + \infty ). $$

Thus, for any $\varepsilon >0$, there exists an integer $n_{0}(\varepsilon )$ such that, for $n>n_{0}(\varepsilon )$,

23 $$ \lambda_{n}\bigl(A_{n}^{*} \bigr)^{\frac{\lambda }{\lambda_{n}}}>(\vartheta - \varepsilon )e\lambda . $$

By Lemma 4.1, it follows from (23) that for $n>n_{0}(\varepsilon )$

24 $$\begin{aligned} \frac{\log M_{u}(\sigma ,F)}{e^{\lambda \sigma }} &\geq \frac{\log A _{n}^{*}+\lambda_{n}\sigma -\log 2}{e^{\lambda \sigma }} \\ & >e^{-\lambda \sigma } \biggl( \lambda_{n}\sigma +\frac{\lambda_{n}}{ \lambda }\log \bigl[(\vartheta -\varepsilon )e\lambda \bigr] -\frac{\lambda_{n}}{ \lambda }\log \lambda_{n}-\log 2 \biggr) . \end{aligned}$$

Let

$$ \biggl( \frac{\lambda_{n}}{\lambda \vartheta } \biggr) ^{\frac{1}{\lambda}}\leq e^{\sigma }< \biggl( \frac{\lambda_{n+1}}{\lambda \vartheta } \biggr) ^{\frac{1}{\lambda }}, $$

and take

$$ \sigma =\frac{1}{\lambda }\log \biggl(\frac{\lambda_{n}}{\lambda \vartheta }\biggr)+o\biggl( \frac{1}{\lambda_{n}}\biggr). $$

Then from (24) it follows

25 $$ \frac{\log M_{u}(\sigma ,F)}{e^{\lambda \sigma }}\geq \frac{\lambda \vartheta }{\lambda_{n+1}} \biggl( \frac{\lambda_{n}}{\lambda }\log \frac{1}{ \lambda \vartheta } +\frac{\lambda_{n}}{\lambda }\log \bigl((\vartheta - \varepsilon )e\lambda \bigr)-\log 2+o(1) \biggr) . $$

Since $\lambda_{n}\sim \lambda_{n+1}$ and $\lambda_{n}\rightarrow + \infty $ as $n\rightarrow +\infty $, thus by a simple computation, from (25) we have $\tau_{\lambda }\geq \vartheta $. When $\vartheta =0$, $\tau_{\lambda }\geq \vartheta $ is obvious; if $\vartheta =\infty $, we also prove that $\tau_{\lambda }\geq \vartheta $ by using the same argument as above. Hence we prove that (11) holds.

Let $\mu (\sigma ,F)$ denote the maximum term for $\Re s=\sigma$, $- \infty < t<+\infty $. Since

$$ \psi (n)=\frac{\log A^{*}_{n}-\log A^{*}_{n+1}}{\lambda_{n+1}-\lambda _{n}} $$

forms a non-decreasing function of *n* for $n>n_{0}$, then for $\psi (n-1)\leq \sigma <\psi (n)$

$$ \log \mu (\sigma ,F)=\log A_{n}^{*}+\lambda_{n} \sigma . $$

Since $\tau_{\lambda }<\infty $, for any small $\varepsilon >0$, it follows from (20) that

26 $$ \log \mu (\sigma ,F)=\log A_{n}^{*}+ \lambda_{n}\sigma \geq ( \tau_{\lambda }-\varepsilon )\exp (\lambda \sigma ) $$

for $\sigma >\sigma_{0}$ and all *n* such that $\psi (n-1)\leq \sigma <\psi (n)$.

Let $\Re s=\sigma >\sigma_{0}$ and $A_{n_{1}}^{*}\exp (\sigma \lambda_{n_{1}})$ and $A_{n_{2}}^{*}\exp (\sigma \lambda_{n_{2}})$ ($n_{1}>n_{0}, \psi (n-1)>\sigma_{0}$) be two consecutive maximum terms such that $n_{2}-1\geq n_{1}$, it follows from (26) that

$$ \log A_{n_{2}}^{*}+\lambda_{n_{2}}\sigma \geq ( \tau_{\lambda }-\varepsilon )\exp (\lambda \sigma ) $$

for all $\sigma >\sigma_{0}$ satisfying $\psi (n_{2}-1)\leq \sigma < \psi (n_{2})$. Let $n_{1}\leq n\leq n_{2}-1$, then

$$ \psi (n_{1})=\psi (n_{1}+1)=\cdots =\psi (n)=\cdots =\psi (n_{2}-1) $$

and $A_{n}^{*}\exp (\lambda_{n}\sigma )=A_{n_{2}}^{*}\exp (\lambda _{n_{2}}\sigma )$ for $\sigma =\psi (n)$. Then there exists a positive integer $n_{1}$ such that, for $n>n_{1}$ and $\sigma >\sigma_{0}$,

$$ \log A_{n}^{*}>(\tau_{\lambda }-\varepsilon )e^{\lambda \sigma }- \lambda_{n}\sigma . $$

Since $e^{x}\geq ex$ for any *x*, so it follows

27 $$ \lambda_{n}\bigl(A_{n}^{*} \bigr)^{\frac{\lambda }{\lambda_{n}}}> \frac{\lambda _{n}}{e^{\lambda \sigma }} \exp \biggl\{ \frac{\lambda (\tau_{\lambda }- \varepsilon )}{\lambda_{n}}e^{\lambda \sigma } \biggr\} >\frac{\lambda _{n}}{e^{\lambda \sigma }}\frac{e(\tau_{\lambda }-\varepsilon )\lambda }{\lambda_{n}}e^{\lambda \sigma } =e( \tau_{\lambda }-\varepsilon ) \lambda . $$

Thus, for $\varepsilon \rightarrow 0$ and $n\rightarrow +\infty $, from (27) it follows

28 $$ \vartheta =\liminf_{n\rightarrow +\infty }\frac{\lambda_{n}}{e\lambda } \bigl(A_{n}^{*}\bigr)^{\frac{\lambda }{\lambda_{n}}}\geq \tau_{\lambda }. $$

Hence, this proves that (12) holds.

### The proof of Theorem 2.5

To prove this theorem, we require the following lemma.

#### Lemma 4.3

*If the abscissa* $\sigma_{u}^{F}=+\infty $ *of uniform convergence of the Laplace*-*Stieltjes transformation* $F(s)$ *and sequence* (2) *satisfies* (4), (6), *then for any real number* *β*, *we have*

$$ \biggl\vert \int_{\lambda_{k}}^{\infty }\exp \bigl\{ (\beta +it)y\bigr\} \,d\alpha (y)\biggr\vert \leq 2\sum_{n=k}^{+\infty }A_{n}^{*} \exp \{\beta \lambda_{n+1}\}, $$

*where*

$$ A_{n}^{*}=\sup_{\lambda_{n}< x\leq \lambda_{n+1},-\infty < t< +\infty } \biggl\vert \int_{\lambda_{n}}^{x}e^{ity}\,d\alpha (y)\biggr\vert . $$

#### Proof

Set

$$ I(x;it)= \int_{0}^{x}\exp \{ity\}\,d\alpha (y). $$

For any real number *β*, since

$$ \biggl\vert \int_{\lambda_{k}}^{\infty }\exp \bigl\{ (\beta +it)y\bigr\} \,d\alpha (y)\biggr\vert =\lim_{b\rightarrow +\infty }\biggl\vert \int_{\lambda_{k}}^{b} \exp \bigl\{ (\beta +it)y\bigr\} \,d \alpha (y)\biggr\vert . $$

Set $I_{j+k}(b;it)=\int_{\lambda_{j+k}}^{b}\exp \{ity\}\,d\alpha (y)$, $(\lambda_{j+k}< b\leq \lambda_{j+k+1})$, then we have $\vert I_{j+k}(b;it) \vert \leq A_{j+k}^{*}$. Thus, it follows

$$\begin{aligned}& \biggl\vert \int_{\lambda_{k}}^{b}\exp \bigl\{ (\beta +it)y\bigr\} \,d \alpha (y)\biggr\vert \\& \quad = \Biggl\vert \sum_{j=k}^{n+k-1} \int_{\lambda_{j}}^{\lambda_{j+1}}\exp \{ \beta y\}d_{y}I_{j}(y;it)+ \int_{\lambda_{n+k}}^{b}\exp \{\beta y\}d _{y}I_{n+k}(y;it) \Biggr\vert \\& \quad = \Biggl\vert \Biggl[ \sum_{j=k}^{n+k-1}e^{\lambda_{j+1}\beta }I_{j}( \lambda_{j+1};it)-\beta \int_{\lambda_{j}}^{\lambda_{j+1}}e^{\beta y}I_{j}(y;it) \,dy\Biggr] \\& \quad \quad {} +e^{\beta b}I_{n+k}(b;it)-\beta \int_{\lambda_{n+k}}^{b}e^{\beta y}I_{j}(y;it) \,dy\Biggr\vert \\& \quad \leq \sum_{j=k}^{n+k-1}\bigl[ A_{j}^{*}e^{\lambda_{j+1}\beta }+A_{j} ^{*} \bigl(e^{\lambda_{j+1}\beta }-e^{\lambda_{j}\beta }\bigr)\bigr] +2e^{\beta \lambda_{n+k+1}}A_{n+k}^{*}-e^{\beta \lambda_{n+k}}A_{n+k}^{*} \\& \quad \leq 2\sum_{j=k}^{n+k} A_{n}^{*}e^{\lambda_{n+1}\beta }. \end{aligned}$$

When $n\rightarrow +\infty $, we have $b\rightarrow +\infty $, thus we have

$$ \biggl\vert \int_{\lambda_{k}}^{\infty }\exp \bigl\{ (\beta +it)y\bigr\} \,d\alpha (y)\biggr\vert \leq 2\sum_{n=k}^{+\infty }A_{n}^{*} \exp \{\beta \lambda_{n+1}\}. $$

 □

Now, we are going to prove Theorem 2.5.

### The proof of Theorem 2.5

Let

$$ \vartheta_{1}= \liminf_{n\rightarrow \infty } \biggl( \frac{\lambda_{n}}{e \lambda } \biggr) \bigl(E_{n-1}(F,\beta )\exp (-\beta \lambda_{n})\bigr)^{\frac{ \lambda }{\lambda_{n}}} \quad (0< \vartheta_{1}< + \infty ). $$

Then, for any small $\varepsilon >0$, there exists an integer $n_{0}(\varepsilon )$ such that, for any $n>n_{0}(\varepsilon )$,

29 $$ \log \bigl(E_{n-1}(F,\beta )\exp (-\beta \lambda_{n})\bigr)>\frac{\lambda_{n}}{ \lambda }\log \frac{(\vartheta_{1}-\varepsilon )e\lambda }{\lambda _{n}}. $$

Since $F(s)\in L_{\infty }$, thus for any constant *β* ($-\infty <\beta <+\infty$), we have $F(s)\in \overline{L}_{\beta }$. For $\beta <\sigma <+\infty $. It follows from the definitions of $E_{n}(F,\beta )$ and $p_{n}$ that

30 $$\begin{aligned} E_{n}(F,\beta ) &\leq \Vert F-p_{n} \Vert _{\beta }\leq \bigl\vert F(\beta +it)-p_{n}(\beta +it) \bigr\vert \\ &\leq \biggl\vert \int_{0}^{+\infty }\exp \bigl\{ (\beta +it)y\bigr\} \,d\alpha (y)- \int_{0}^{\lambda_{n}}\exp \bigl\{ (\beta +it)y\bigr\} \,d \alpha (y)\biggr\vert \\ &=\biggl\vert \int_{\lambda_{n}}^{\infty }\exp \bigl\{ (\beta +it)y\bigr\} \,d\alpha (y)\biggr\vert . \end{aligned}$$

Thus, from the definition of $A_{n}^{*}$ and $M_{u}(\sigma ,F)$, and by Lemma 4.1, we have $A_{n}^{*}\leq 2 M_{u}(\sigma ,F)e^{-\sigma \lambda _{n}}$ for any *σ* ($\beta <\sigma <+\infty$). It follows from (30) and Lemma 4.3 that

31 $$ E_{n}(F,\beta )\leq 2\sum_{k=n+1}^{\infty }A_{k-1}^{*} \exp \{\beta \lambda_{k}\}\leq 4M_{u}(\sigma ,F)\sum _{k=n+1}^{\infty }\exp \bigl\{ ( \beta -\sigma ) \lambda_{k}\bigr\} . $$

From (4), take $h'$ ($0< h'< h$) such that $(\lambda_{n+1} -\lambda_{n}) \geq h'$ for $n\geq 0$. Then, for $\sigma \geq \frac{\beta }{2}$, it follows from (31) that

$$\begin{aligned} E_{n}(F,\beta ) &\leq 4M_{u}(\sigma ,F)\exp \bigl\{ \lambda_{n+1}(\beta - \sigma )\bigr\} \sum_{k=n+1}^{\infty } \exp \bigl\{ (\lambda_{k}-\lambda_{n+1}) ( \beta -\sigma )\bigr\} \\ &\leq 4M_{u}(\sigma ,F)\exp \bigl\{ \lambda_{n+1}(\beta - \sigma )\bigr\} \exp \biggl\{ -\frac{ \beta }{2}h'(n+1)\biggr\} \sum _{k=n+1}^{\infty }\biggl(\exp \biggl\{ \frac{\beta }{2}h'k \biggr\} \biggr) \\ &=4M_{u}(\sigma ,F)\exp \bigl\{ \lambda_{n+1}(\beta -\sigma ) \bigr\} \biggl( 1- \exp \biggl\{ \frac{\beta }{2}h'\biggr\} \biggr) ^{-1}, \end{aligned}$$

that is,

32 $$ E_{n-1}(F,\beta )\leq KM_{u}(\sigma ,F)\exp \bigl\{ \lambda_{n}(\beta - \sigma )\bigr\} , $$

where *K* is a constant. Let

$$ \gamma_{n}= E_{n-1}(F,\beta )\exp (-\beta\lambda_{n})\quad (n=1,2,\ldots ). $$

Thus, from (29) and (32), it follows that for $n>n_{0}(\varepsilon )$

33 $$\begin{aligned} \frac{\log M_{u}(\sigma ,F)}{e^{\lambda \sigma }} &\geq \frac{\log \gamma_{n}+\lambda_{n}\sigma -\log K}{e^{\lambda \sigma }} \\ & >e^{-\lambda \sigma } \biggl( \lambda_{n}\sigma +\frac{\lambda_{n}}{ \lambda }\log \bigl[(\vartheta_{1}-\varepsilon )e\lambda \bigr]-\frac{\lambda _{n}}{\lambda } \log \lambda_{n}-\log K \biggr) . \end{aligned}$$

By using the same argument as in Theorem 2.4, we can easily prove that $\tau_{\lambda }\geq \vartheta_{1}$.

From the proof of Theorem 2.4, we have that there exists a positive integer $n_{1}$ such that

$$ \log A_{n}^{*}>(\tau_{\lambda }-\varepsilon )e^{\lambda \sigma }- \lambda_{n}\sigma $$

for $n>n_{1}$ and $\sigma >\sigma_{0}$. Since for any $\beta <+\infty $, from the definition of $E_{k}(F,\beta )$, there exists $p_{1} \in \Pi_{n-1}$ such that

34 $$ \Vert F-p_{1} \Vert \leq 2E_{n-1}(F,\beta ). $$

And since

$$\begin{aligned} A_{n}^{*}\exp \{\beta \lambda_{n}\} & = \sup_{\lambda_{n}< x\leq \lambda_{n+1},-\infty < t< +\infty }\biggl\vert \int _{\lambda_{n}}^{x}\exp \{ity\}\,d\alpha (y)\biggr\vert \exp \{\beta \lambda _{n}\} \\ &\leq \sup_{\lambda_{n}< x\leq \lambda_{n+1},-\infty < t< + \infty }\biggl\vert \int_{\lambda_{n}}^{x}\exp \bigl\{ (\beta +it)y\bigr\} \,d\alpha (y)\biggr\vert \\ &\leq \sup_{-\infty < t< +\infty }\biggl\vert \int_{\lambda_{n}}^{ \infty }\exp \bigl\{ (\beta +it)y\bigr\} \,d \alpha (y)\biggr\vert , \end{aligned}$$

thus for any $p\in \Pi_{n-1}$, it follows

35 $$ A_{n}^{*}\exp \{\beta \lambda_{n} \}\leq \bigl\vert F(\beta +it)-p(\beta +it) \bigr\vert \leq \Vert F-p \Vert _{\beta }. $$

Hence from (34) and (35), for any $\beta <+\infty $ and $F(s)\in L _{\infty }$, we have

$$ A_{n}^{*}\exp \{\beta \lambda_{n}\}\leq 2E_{n-1}(F,\beta ). $$

Since $e^{x}\geq ex$ for any *x*, so it follows

36 $$\begin{aligned} \lambda_{n}(\gamma_{n})^{\frac{\lambda }{\lambda_{n}}}&> \frac{\lambda _{n}}{e^{\lambda \sigma }} \exp \biggl\{ \frac{\lambda (\tau_{\lambda }- \varepsilon )}{\lambda_{n}}e^{\lambda \sigma }- \frac{\lambda \log 2}{ \lambda_{n}} \biggr\} \\ &>\frac{\lambda_{n}}{e^{\lambda \sigma }} \biggl( \frac{e(\tau_{\lambda }-\varepsilon )\lambda }{\lambda_{n}}e^{\lambda \sigma }\exp \bigl\{ o(1) \bigr\} \biggr) =e(\tau_{\lambda }-\varepsilon )\lambda . \end{aligned}$$

Thus, for $\varepsilon \rightarrow 0$ and $n\rightarrow +\infty $, from (36) it follows

$$ \vartheta_{1}=\liminf_{n\rightarrow \infty }\frac{\lambda_{n}}{e \lambda }( \gamma_{n})^{\frac{\lambda }{\lambda_{n}}}\geq \tau_{\lambda }. $$

Since $[E_{n-1}(F,\beta )\exp (-\beta \lambda_{n})]^{\frac{\lambda }{ \lambda_{n}}}=[E_{n-1}(F,\beta )]^{\frac{\lambda }{\lambda_{n}}} \exp (-\beta \lambda )$, then (14) follows.

Therefore, we complete the proof of Theorem 2.5.

### Remarks

From the proof of Theorem 2.5, and combining those results of the Laplace-Stieltjes transforms in Ref. [14, 16, 27], we can obtain the following results on the approximation of Laplace-Stieltjes transforms, which can be found partly in [28].

#### Theorem 4.1

*If the L*-*S transform* $F(s)\in L_{\infty }$ *and is of order* *ρ* ($0<\rho <\infty$) *and of type* *T*, *then for any real number* $-\infty < \beta <+\infty $, *we have*

$$ \rho =\limsup_{n\rightarrow +\infty }\frac{\lambda_{n}\log \lambda _{n}}{-\log E_{n-1}(F,\beta )\exp (-\beta \lambda_{n})}= \limsup _{n\rightarrow +\infty }\frac{\lambda_{n}\log \lambda_{n}}{- \log E_{n-1}(F,\beta )} $$

*and*

$$ \begin{aligned} T&=\limsup_{n\rightarrow +\infty }\frac{\lambda_{n}}{\rho e}\bigl(E_{n-1}(F, \beta )\exp (-\beta \lambda_{n})\bigr)^{\frac{\rho }{\lambda_{n}}} \\ &= \limsup _{n\rightarrow +\infty }\frac{\lambda_{n}}{\rho \exp (\rho \beta +1)}\bigl(E_{n-1}(F,\beta ) \bigr)^{\frac{\rho }{\lambda_{n}}}. \end{aligned} $$

*Furthermore*, *if* $F(s)$ *is of the lower order* *λ* *and the lower type* *τ*, *and* $\lambda_{n}\sim \lambda_{n+1}$ *and the function*

$$ \psi (n)=\frac{\log A^{*}_{n}-\log A^{*}_{n+1}}{\lambda_{n+1}-\lambda _{n}} $$

*forms a non*-*decreasing function of* *n* *for* $n>n_{0}$, *then we have*

$$ \lambda =\liminf_{n\rightarrow +\infty }\frac{\lambda_{n}\log \lambda _{n}}{-\log E_{n-1}(F,\beta )}, \qquad \tau = \liminf _{n\rightarrow + \infty }\frac{\lambda_{n}}{\rho \exp (\rho \beta +1)}\bigl(E_{n-1}(F, \beta ) \bigr)^{\frac{\rho }{\lambda_{n}}}. $$

#### Theorem 4.2

*If the L*-*S transform* $F(s)\in L_{\infty }$, *then for any real number* $-\infty <\beta <+\infty $. *For* $p=1$, *we have*

$$ \limsup_{\sigma \rightarrow +\infty }\frac{h(\log M_{u}(\sigma ,F))}{h( \sigma )}-1= \limsup _{n\rightarrow +\infty }\frac{h(\lambda_{n})}{h ( -\frac{1}{\lambda_{n}} \log [E_{n-1}(F,\beta )\exp (-\beta \lambda_{n})] ) }, $$

*and for* $p=2,3,\ldots $ , *we have*

$$\begin{aligned} &\limsup_{n\rightarrow +\infty }\frac{h(\lambda_{n})}{h ( -\frac{1}{ \lambda_{n}} \log [E_{n-1}(F,\beta )\exp (-\beta \lambda_{n})] ) } \\ &\quad \leq \limsup _{\sigma \rightarrow +\infty }\frac{h(\log M_{u}(\sigma ,F))}{h( \sigma )} \\ &\quad \leq \limsup_{n\rightarrow +\infty }\frac{h(\lambda_{n})}{h ( -\frac{1}{ \lambda_{n}} \log [E_{n-1}(F,\beta )\exp (-\beta \lambda_{n})] ) }+1, \end{aligned}$$

*where* $h(x)$ *satisfies the following conditions*:

(i) $h(x)$ *is defined on* $[a,+\infty )$ *and is positive*, *strictly increasing*, *differentiable and tends to* +∞ *as* $x\rightarrow + \infty $;
(ii) $\lim_{x\rightarrow +\infty } \frac{d(h(x))}{d(\log^{[p]}x)}=k\in (0,+\infty )$, $p\geq 1, p\in \mathbb{N}^{+}$, *where* $\log^{[0]}x=x, \log^{[1]}x=\log x$ *and* $\log^{[p]}x=\log (\log^{[p-1]}x)$.
